# Clearance of autophagy-associated dying retinal pigment epithelial cells – a possible source for inflammation in age-related macular degeneration

**DOI:** 10.1038/cddis.2016.133

**Published:** 2016-09-08

**Authors:** M Szatmári-Tóth, E Kristóf, Z Veréb, S Akhtar, A Facskó, L Fésüs, A Kauppinen, K Kaarniranta, G Petrovski

**Affiliations:** 1 Department of Biochemistry and Molecular Biology and MTA-DE Stem cell, Apoptosis and Genomics Research Group, University of Debrecen, Debrecen, Hungary; 2 Stem Cells and Eye Research Laboratory, Department of Ophthalmology, Faculty of Medicine, University of Szeged, Szeged, Hungary; 3 Department of Optometry, College of Applied Medicine, King Saud University, Riyadh, Saudi Arabia; 4 School of Pharmacy, Facility of Health Science, University of Eastern Finland, Kuopio, Finland; 5 Centre of Eye Research, Department of Ophthalmology, Oslo University Hospital, University of Oslo, Oslo, Norway

## Abstract

Retinal pigment epithelial (RPE) cells can undergo different forms of cell death, including autophagy-associated cell death during age-related macular degeneration (AMD). Failure of macrophages or dendritic cells (DCs) to engulf the different dying cells in the retina may result in the accumulation of debris and progression of AMD. ARPE-19 and primary human RPE cells undergo autophagy-associated cell death upon serum depletion and oxidative stress induced by hydrogen peroxide (H_2_O_2_). Autophagy was revealed by elevated light-chain-3 II (LC3-II) expression and electron microscopy, while autophagic flux was confirmed by blocking the autophago-lysosomal fusion using chloroquine (CQ) in these cells. The autophagy-associated dying RPE cells were engulfed by human macrophages, DCs and living RPE cells in an increasing and time-dependent manner. Inhibition of autophagy by 3-methyladenine (3-MA) decreased the engulfment of the autophagy-associated dying cells by macrophages, whereas sorting out the GFP-LC3-positive/autophagic cell population or treatment by the glucocorticoid triamcinolone (TC) enhanced it. Increased amounts of IL-6 and IL-8 were released when autophagy-associated dying RPEs were engulfed by macrophages. Our data suggest that cells undergoing autophagy-associated cell death engage in clearance mechanisms guided by professional and non-professional phagocytes, which is accompanied by inflammation as part of an *in vitro* modeling of AMD pathogenesis.

The human retina is under constant remodeling throughout the lifetime, with various forms of cell death occurring in its 10 anatomical layers including the outermost – retinal pigment epithelium (RPE).^1^ Autophagic cell death has been described as early as embryonic development and organogenesis^2^ and as late as old age, in particular, in neurodegenerative diseases.^3^ The retina – being an extension of the central nervous system into the eye – is also prone to autophagy and degeneration with age-related macular degeneration (AMD) being the leading cause of legal blindness in the aging population of the Western countries.^4^

Corticosteroids are commonly used in ophthalmology for treatment of various retinal diseases.^5^ Triamcinolone (TC), a conventional corticosteroid with anti-inflammatory and anti-angiogenic activity, is a potent treatment for intraocular proliferative, edematous and neovascular ocular diseases^6, 7^ and AMD,^8, 9^ in particular, exudative AMD.^10, 11^ TC treatment can be used alone or in combination with other treatments such as photodynamic therapy with verteporfin or anti-vascular endothelial growth factor agents.^12^

Since the first description of autophagy in 1966,^13, 14^ the process has been ascribed to have a role as survival mechanism under poor nutritional conditions.^15^ However, it is now clearly evident that autophagy has a dual role.^16, 17, 18^ This degradative mechanism for long-lived proteins and damaged organelles via the autophago–lysosomal pathway can provide possibility of cellular self-destruction under chronic stress conditions.^19, 20^ RPE cells can also be induced to undergo autophagy-associated cell death by starvation and oxidative stress.^21, 22, 23^

The final fate of dead cells in the body depends upon the clearance mechanisms posed by macrophages and dendritic cells (DCs) both acting as professional phagocytes and/or antigen-presenting cells.^24^ These cells are capable of engulfing apoptotic and necrotic cells without causing inflammation, respectively,^25^ while autophagy-associated dying cells are capable of inducing inflammation.^26, 27, 28^ During embryonic development, clearance of a large number of apoptotic cells takes place; similarly, clearance of apoptotic granulocytes occurs during inflammation, and daily clearance of photoreceptor outer segments occurs throughout the lifetime^29, 30^ and intensifies during aging.^31, 32^ Many different cell types are equipped with machinery to engulf, including epithelial cells and RPEs, which can act as non-professional phagocytes.^33^

AMD can be classified in a simplified way as dry, when the Bruch's membrane is still intact,^34^ and wet, when choroidal neovascularizations (CNVs) penetrate through the membrane and many cells present in the blood circulation can reach the damaged area.^35, 36^

Autophagy markers in the RPEs have been detected in cadaver eyes from AMD patients.^37, 38, 39, 40^ To our present knowledge, the final fate and clearance mechanism of cells dying through an autophagy-associated process in the retina have not been revealed. We have initiated a series of experiments, in which autophagy-associated cell death was induced in ARPE-19 and primary human RPE (hRPE) cells by serum deprivation and oxidative stress by H_2_O_2_. The engulfment of these cells by professional or non-professional phagocytes, human macrophages, DCs or RPEs, respectively, was studied accordingly. Furthermore, the effect of TC, a glucocorticoid which we recently described to enhance phagocytosis of anoikic dying RPEs,^41^ was studied upon engulfment of autophagy-associated dying RPEs. We show here that autophagy-associated dying RPEs are engulfed by macrophages, DCs and RPE cells in an increasing and time-dependent manner. This process is accompanied by a pro-inflammatory response, while TC enhances the engulfment capacity of macrophages. Altogether, the data contribute to better understanding and *in vitro* modeling of AMD pathogenesis and its possible implications in the search for treatment targets.

## Results

### Serum deprivation and H_2_O_2_ co-treatment induce autophagy in RPE cells

The induction of autophagy by serum deprivation and H_2_O_2_ co-treatment (both being previously described as inducers of autophagy) was studied in RPE cells^23, 42, 43^ and in other cell types.^44, 45, 46^

**Figure 1:**
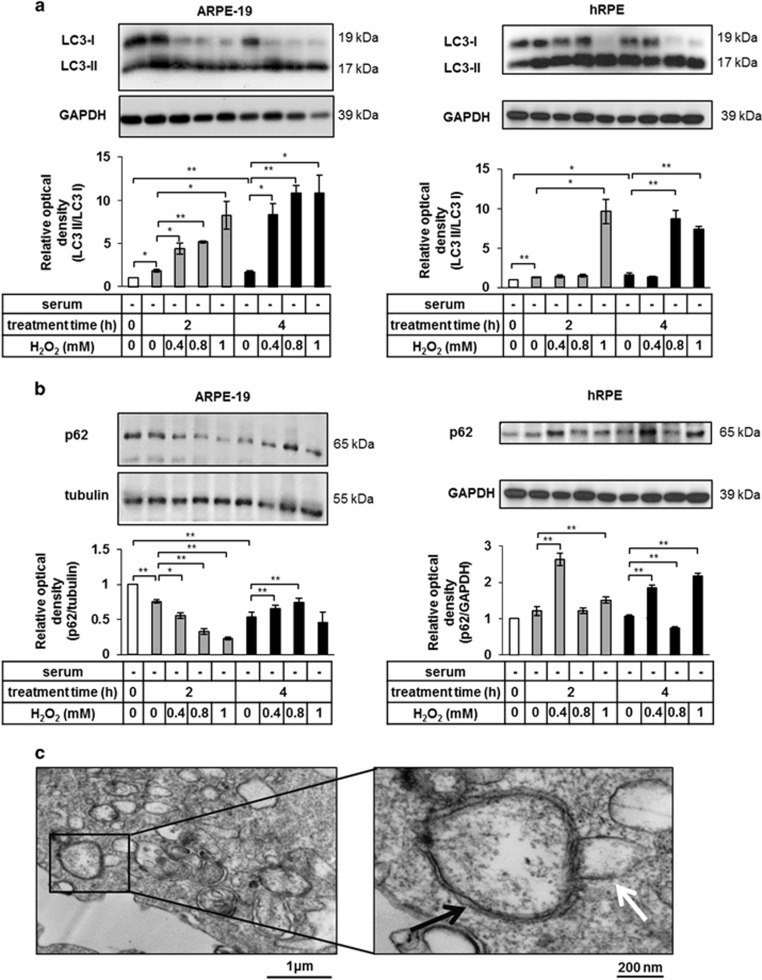
Serum deprivation and H_2_O_2_ co-treatment result in induced autophagy in ARPE-19 and human primary RPE (hRPE) cells. Detection of autophagy by quantification of the LC3-II/LC3-I ratio (**a**) and p62 expression (**b**) in ARPE-19 (left panels) and primary hRPE cells (right panels) using western blot analysis after increasing time (2 h, 4 h) and concentrations (0.4 mM, 0.8 mM, 1 mM) of H_2_O_2_-treatment in the absence of serum. Relative optical density was determined by densitometry using the ImageJ software (white bars show the untreated controls; gray and black bars represent 2 h and 4 h long treatments, respectively). GAPDH and tubulin were used as loading controls. Data are mean±S.E.M. of three independent measurements, *P<0.05, **P<0.01. (**c**) Double-membraned autophagic vesicles (black arrow) were detected in H_2_O_2_-treated (2 h, 1 mM) ARPE-19 cells by transmission electron microscopy (TEM, Philips CM 10 microscope). Fusion between the autophagosomes and lysosomes (white arrow) was observed. Scale bars represent 1 *μ*m (left panel) or 200 nm (right panel). Data are representative of three independent experiments

Time- and concentration-dependent induction of autophagy was determined by western blot analysis of LC3 expression. In both, ARPE-19 and hRPE cells, the ratio of 17 kDa LC3-II (the autophagosomal membrane-bound form of LC3) and 19 kDa LC3-I (the free cytosolic form) increased the most at 2 h of serum deprivation and 1 mM H_2_O_2_ treatment (Figure 1a), while the SQSTM1/p62 expression (additional autophagosomal membrane-associated marker for detecting autophagy)^47^ showed decreasing tendency under the same treatment modality in ARPE-19 cells (Figure 1b). Autophagic vacuoles (AVs) were detected by transmission electron microscopy (TEM) at 2 h of 1 mM H_2_O_2_ treatment. The presence of double-membraned AVs containing cytosolic components (black arrow), some of which were being fused with the lysosomes (white arrow), could also be confirmed (Figure 1c).

### Increased autophagy in RPE cells is accompanied by increased autophagic flux

**Figure 2:**
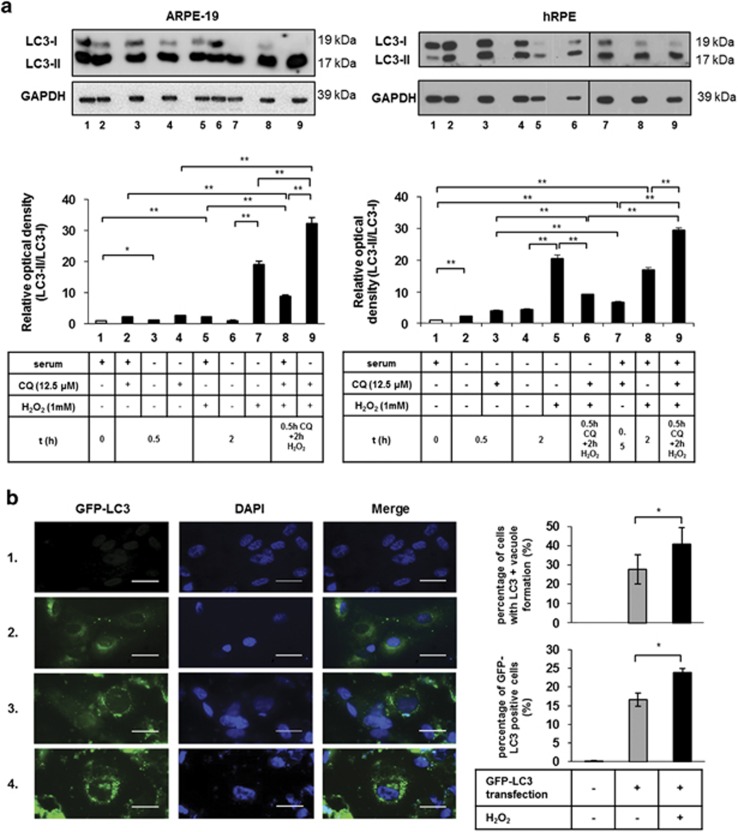
Detection of increased autophagic flux in ARPE-19 and primary hRPE cells. (**a**) Autophagic flux was assessed by western blot analysis based on the quantification of LC3-II/LC3-I ratio. ARPE-19 (left panel) and primary hRPE cells (right panel) were pre-treated with chloroquine (CQ) (0.5 h,12.5 *μ*M) and then treated with H_2_O_2_ (2 h, 1 mM) in the presence or absence of serum. GAPDH was used as a loading control. Relative optical density was determined by densitometry using the ImageJ software. Data are mean±S.E.M. of three independent experiments, *P<0.05, **P<0.01. (**b**) Representative immunofluorescence images of ARPE-19 cells transiently transfected with a GFP-LC3 plasmid. Non-transfected, GFP-LC3-transfected and untreated cells are shown in row 1 and 2, respectively. H_2_O_2_ treatment resulted in the accumulation of perinuclear, ring-shaped GFP-LC3-positive aggregates in the transfected ARPE-19 cells (row 3). CQ and subsequent H_2_O_2_ treatment (2 h, 1 mM) led to more abundant and bigger GFP-LC3-positive AVs (row 4). Cell nuclei were labeled with DAPI. Scale bar represents 20 *μ*m. Images are representative of three independent experiments. Quantification of cells containing GFP-LC3-positive vacuoles (top graph) was performed by manual cell counting based on the fluorescent images. The ratio of the number of GFP-LC3-positive cells to the total cell number in H_2_O_2_-treated ARPE-19 cells is shown as a percentage. Data are expressed as mean±S.D. of at least 10 different visual fields on microscopy from three independent experiments in each condition (*P<0.05, by Student *t*-test). The percentage of GFP-LC3-positive cells (bottom graph) was quantified using FACS analysis. Data are expressed as mean±S.D. of three independent experiments, *P<0.05 by Student *t*-test

The lysosomal inhibitor CQ was used to measure the endogenous LC3-II turnover. The induction of autophagosome formation could be determined by immunoblotting, showing increased LC3-II expression after CQ treatment.^48^ Inhibition of the autophago-lysosomal fusion by CQ significantly increased the LC3-II/LC3-I ratio, and thus autophagic flux was present in H_2_O_2_-treated ARPE-19 and hRPE cells (Figure 2a).

Induction of autophagy in ARPE-19 cells led to the accumulation of perinuclear green fluorescent protein (GFP)-LC3-positive aggregates or ring-shaped AVs, which could be detected by fluorescence microscopy (Figure 2b). The number and size of the GFP-LC3-positive AVs peaked at 2 h of 1 mM H_2_O_2_ treatment; more abundant and bigger GFP-LC3-positive AVs were found as a result of the CQ treatment (40.9±8.4% of the cells contained GFP-LC3-positive vacuoles counted manually and 23.9±1.2% of the cells were GFP-LC3-positive when quantified by fluorescence-activated cell sorter (FACS) analysis).

### Induction of autophagy-associated cell death in RPE cells

**Figure 3:**
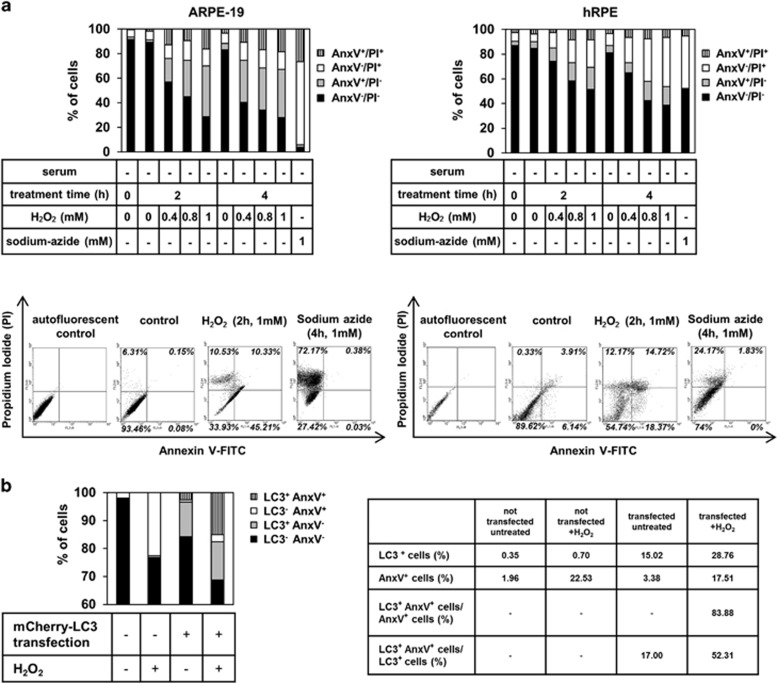
ARPE-19 and primary hRPE cells die as a result of serum deprivation and H_2_O_2_ co-treatment in a time- and concentration-dependent manner. (**a**) Quantification of the cell death rate by flow cytometry after increasing time intervals (2 h, 4 h) and concentrations (0.4 mM, 0.8 mM, 1 mM) of H_2_O_2_-treatment in the absence of serum in ARPE-19 (left panel) and hRPE (right panel) cells using Annexin V (AnxV)-FITC/propidium iodide (PI) labeling. Sodium-azide treatment (4 h, 1 mM) was used as positive control for necrotic cell death. The bar charts indicate the percentage of AnxV^−^/PI^−^ (viable; black bars), AnxV^+^/PI^−^ (early apoptotic; gray bars), AnxV^−^/PI^+^ (necrotic; white bars) and AnxV^+^/PI^+^ (late apoptotic; striped bars) cells. Data are shown from three and four independent experiments for ARPE-19 and hRPE, respectively. Representative dot plots of AnxV/PI measurements of dying ARPE-19 and hRPE cells are also shown. The horizontal axis represents intensity of staining for Annexin V (logarithmic scale) and the vertical axis shows intensity of staining for PI (logarithmic scale). The numbers in the quadrants indicate the percentage of different cell populations: viable (lower left), apoptotic (lower right), necrotic (upper left), late apoptotic cells (upper right). Data are representative of three independent experiments. (**b**) The rate of H_2_O_2_-induced cell death of ARPE-19 cells containing LC3-positive AVs and showing PS externalization is demonstrated; mCherry-LC3 plasmid was transiently transfected into ARPE-19 cells using PEI reagent. The cells were treated with H_2_O_2_ (2 h, 1mM) and then annexin V-FITC labeling was performed. The bar charts represent the percentage of LC3^−^AnxV^−^ (black bars), LC3^+^AnxV^−^ (gray bars), LC3^−^AnxV^+^ (white bars) and LC3^+^AnxV^+^ (striped bars) cells. The table shows the percentage of LC3^+^ and AnxV^+^ cells and the rate of LC3^+^AnxV^+^/AnxV^+^ and LC3^+^AnxV^+^/LC3^+^ untreated and H_2_O_2_-treated cells. Data are shown from three independent experiments

ARPE-19 and hRPE cells die because of serum deprivation and H_2_O_2_ co-treatment in a time- and concentration-dependent manner as demonstrated by a high-throughput flow cytometry-based method.^49^ Cells that are viable are both Annexin V (AnxV)- and propidium iodide (PI)-negative, while cells that are AnxV^+^ and PI^−^, AnxV^−^ and PI^+^ indicate early apoptosis and necrosis, respectively. In addition, AnxV/PI double positivity is a sign of late apoptosis, primary or secondary necrosis.^50, 51^ After 2 h of 1 mM H_2_O_2_ treatment, the percentage of living ARPE-19 cells, compared with the untreated control ones, significantly decreased from 91.4±1.7% to 28.6±14.2%. In case of hRPE cells, the ratio of living cells changed from 87.1±4.9% to 51.6±3.6%. In parallel, the percentage of only AnxV^+^ ARPE-19 cells increased from 2.1±2% to 41±10.8% 17.7±12.7% of the hRPE cells became AnxV^+^ as a result of induction of autophagy-associated cell death, while the untreated control contained only 3.2±2.8% of these early apoptotic cells (Figure 3a). Altogether, the H_2_O_2_ treatment causes RPE cells to undergo a mixture of different cell death modalities over the tested time.

ARPE-19 cells were transiently transfected with mCherry-LC3 plasmid, then treated with H_2_O_2_ (2 h, 1 mM); accordingly, the percentage of LC3^+^ untreated ARPE-19 cells was 15.02% and it increased to 28.76% upon H_2_O_2_ treatment; meanwhile, the percentage of AnxV^+^ cells increased from 3.4 to 17.5%. Moreover, 52.3% of LC3^+^ cells were AnxV^+^, while 83.88% of AnxV^+^ cells were LC3^+^ as well (Figure 3b). These data suggest that autophagy-associated process was induced in most of the dying ARPE-19 cells as a result of H_2_O_2_ treatment.

### Autophagy-associated dying RPE cells are efficiently engulfed by macrophages, DCs and non-dying RPE cells

**Figure 4:**
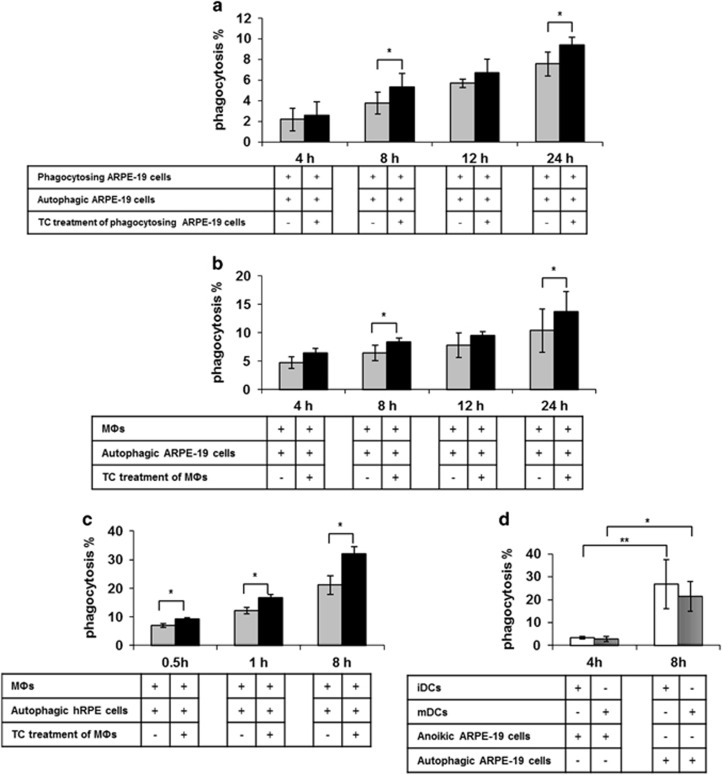
Non-professional and professional phagocytes are able to efficiently engulf autophagy-associated dying RPE cells *in vitro*. The clearance of autophagy-associated dying ARPE-19 cells by living ARPE-19 cells (**a**) and macrophages (MΦ) (**b**) after increasing co-incubation periods (4 h, 8 h, 12 h, 24 h) is shown as determined by FACS analysis. Phagocytes pre-treated with triamcinolone (TC) (48 h, 1 *μ*M) were labeled by black bars (gray bars indicate untreated phagocytes). (**c**) The rate of phagocytosis of autophagy-associated dying primary hRPE cells by MΦs treated with (black bars) or without (gray bars) TC were measured after increasing co-incubation periods (0.5 h, 1 h, 8 h) by FACS analysis. (**d**) Phagocytic capacity of immature DCs (iDCs) (white bars) and mature DCs (mDCs) (striped bars) for engulfment of anoikic or autophagy-associated dying ARPE-19 cells after 4 and 8 h co-incubation is shown, respectively. Bars represent the mean±S.D. of four independent experiments. *P<0.05, **P<0.01

Although phagocytosis of apoptotic-, autophagy-associated dying and necrotic cells has been extensively studied in other organ systems, and we have previously shown the clearance dynamics of apoptotic/anoikic RPE cells *in vitro,*^41^ no data exist on how autophagy-associated dying cells get removed from the retina. Living RPE cells, macrophages and DCs could engulf autophagy-associated dying RPE cells with increasing number of phagocytes containing cell corpses over a 24h period quantified by flow cytometry (Figures 4a–d and Supplementary Figure 1) and demonstrated by time-lapse microscopy (Supplementary Videos 1 and 2).

Living ARPE-19 cells removed autophagy-associated dying ARPE-19 cells in an efficient manner, reaching an average phagocytosis frequency of 3.8±1.1% (Figure 4a) at 8 h of co-incubation. Similarly, the rate of phagocytosis of autophagy-associated dying ARPE-19 cells by macrophages was 6.9±1.7% (Figure 4b). The engulfment of autophagy-associated dying primary hRPE cells by macrophages was even more efficient, the phagocytic capacity being 21.2±3.3% (Figure 4c) after 8 h of co-incubation. TC treatment further enhanced the engulfment process under most conditions (Figures 4a–c and Supplementary Videos 3 and 4). Interestingly, DCs engulfed autophagy-associated dying ARPE-19 cells more effectively: the phagocytic rate was 26.7±10.8% in case of immature DCs (iDCs), and 21.4±6.4% when DCs were activated by IL-1β (5 ng/ml), IL-6 (100 ng/ml), TNF*α* (10 ng/ml), PGE2 (1 *μ*g/ml), granulocyte-macrophage colony-stimulating factor (GMCSF) (80 ng/ml)^52^ (Figure 4d) after 8 h of co-incubation.

### Inhibition of autophagy in ARPE-19 cells by 3-MA attenuates phagocytosis by macrophages

**Figure 5:**
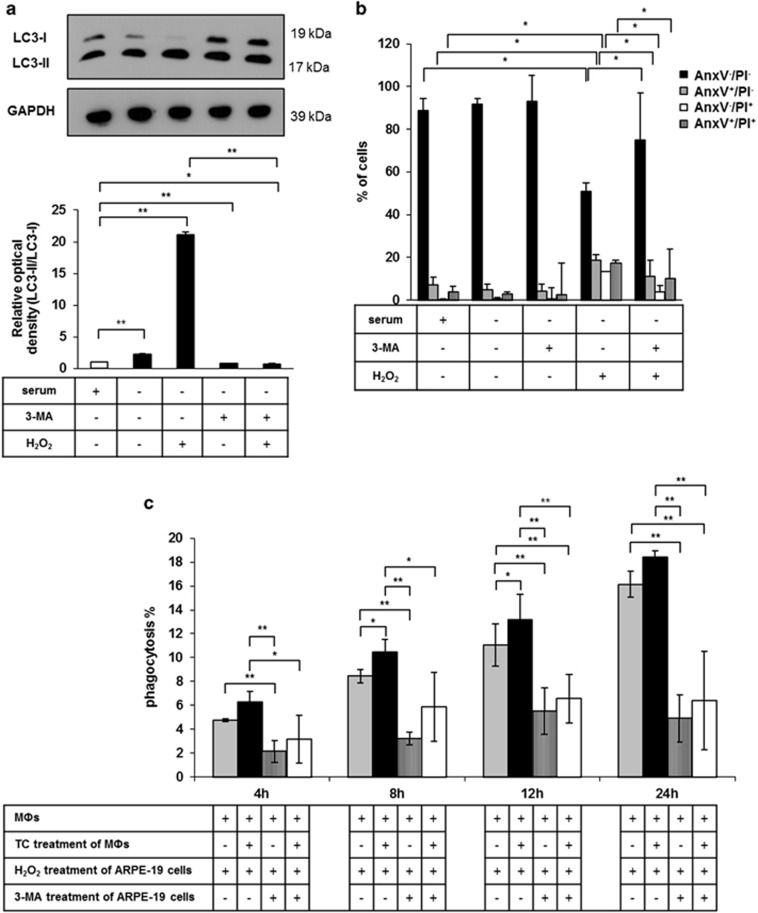
Inhibition of autophagy-associated cell death of ARPE-19 cells by 3-MA and subsequent effect upon phagocytosis. (**a**) Detection of autophagy inhibition by quantification of LC3-II/LC3-I ratio in 3-MA pre-treated (24 h, 10 mM) and H_2_O_2_-treated (2 h, 1 mM) ARPE-19 cells is shown using western blot analysis. Relative optical density was determined by densitometry using the ImageJ software (white bar shows the untreated control, black bars represent the treated samples). GAPDH was used as a loading control. Data are mean±S.E.M. of three independent measurements, *P<0.05, **P<0.01. (**b**) Quantification of the cell death rate of 3-MA pre-treated (24 h, 10 mM) and H_2_O_2_-treated (2 h, 1 mM) ARPE-19 cells by FACS analysis using Annexin V-FITC/PI labeling. Data are expressed as mean±S.D. of four independent experiments, *P<0.05 by Student *t*-test. (**c**) Phagocytosis of 3-MA pre-treated (24 h, 10 mM) and H_2_O_2_-treated (2 h, 1 mM) ARPE-19 cells by untreated and TC-treated (48 h, 1 *μ*M) MФs after increasing co-incubation periods (4 h, 8 h, 12 h, 24 h). Bars represent mean±S.D. of three independent experiments, *P<0.05, **P<0.01

Next, we investigated the effect of 3-MA on cell death and consequently the engulfment of 3-MA/autophagy inhibited ARPE-19 cells; 10 mM 3-MA pre-treatment for 24 h^27^ partially blocked the conversion of LC3-I to LC3-II, which proved that 3-MA could inhibit the autophagic process in H_2_O_2_-treated ARPE-19 cells (Figure 5a). In addition, this inhibitor significantly increased the number of living cells, while it significantly decreased the number of AnxV^+^ and PI^+^, as well as double positive H_2_O_2_-treated ARPE-19 cells. Thus, 3-MA pre-treatment inhibited the autophagy-associated cell death of ARPE-19 cells (Figure 5b). The inhibition of autophagy in ARPE-19 cells by 3-MA significantly decreased the engulfment of autophagy-associated dying cells by untreated and TC-treated macrophages (Figure 5c).

### Engulfment of GFP-LC3-positive/autophagy-associated dying ARPE-19 cells

**Figure 6:**
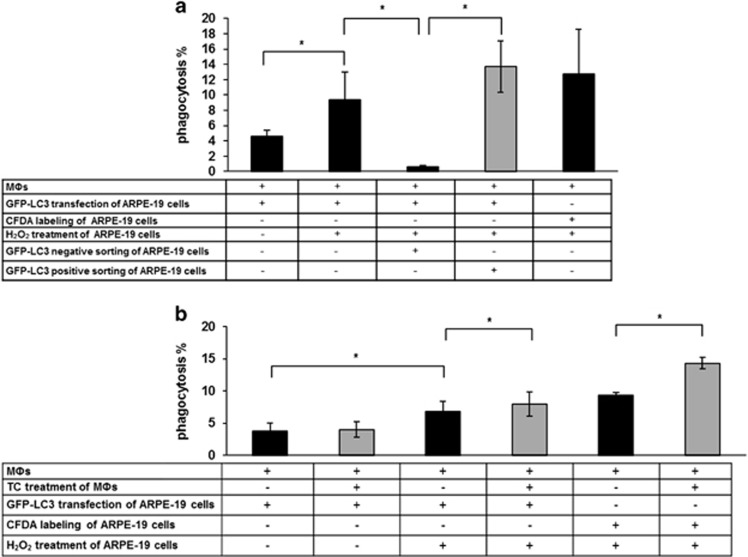
Dynamics of the engulfment of GFP-LC3 transfected, H_2_O_2_-treated ARPE-19 cells. (**a**) Phagocytosis of GFP-LC3-transfected, untreated or H_2_O_2_-treated (2 h, 1 mM) and GFP-LC3-negative or -positive sorted cells, and not transfected, H_2_O_2_-treated ARPE-19 cells by MФs after 8 h co-incubation. (**b**) Clearance of GFP-LC3-transfected, untreated or H_2_O_2_-treated and not transfected, H_2_O_2_-treated ARPE-19 cells by untreated and TC-treated (48 h, 1 *μ*M) MФs after 8 h co-incubation. Bars represent mean±S.D. of three independent experiments, *P<0.05

To determine whether the engulfed cells were actually the autophagy-associated dying cells and not any other type of dying cells, the engulfment of GFP-LC3 transfected, H_2_O_2_-treated ARPE-19 cells was quantified. The actual phagocytic rate was 9.3±3.7%, comparable with the engulfment when vitally stained, H_2_O_2_-treated ARPE-19 cells were co-incubated with the macrophages. In addition, the GFP-LC3-positive and -negative RPE cells were sorted out and co-incubated with macrophages for 8 h. The rate of phagocytosis of non-autophagic, GFP-LC3-negative RPEs was negligible (0.6±0.2%), whereas in contrast, macrophages engulfed the GFP-LC3-positive RPEs at the same rate (13.7±3.3%) (Supplementary Video 7) as they did the non-sorted autophagy-associated dying cells (Figure 6a and Supplementary Video 5), thus confirming their fate in the engulfment process. In addition, TC treatment significantly enhanced the engulfment of GFP-LC3-transfected, H_2_O_2_-treated ARPE-19 cells (Figure 6b and Supplementary Video 6).

### Macrophages engulfing H_2_O_2_-induced dying RPE cells release IL-6 and IL-8

**Figure 7:**
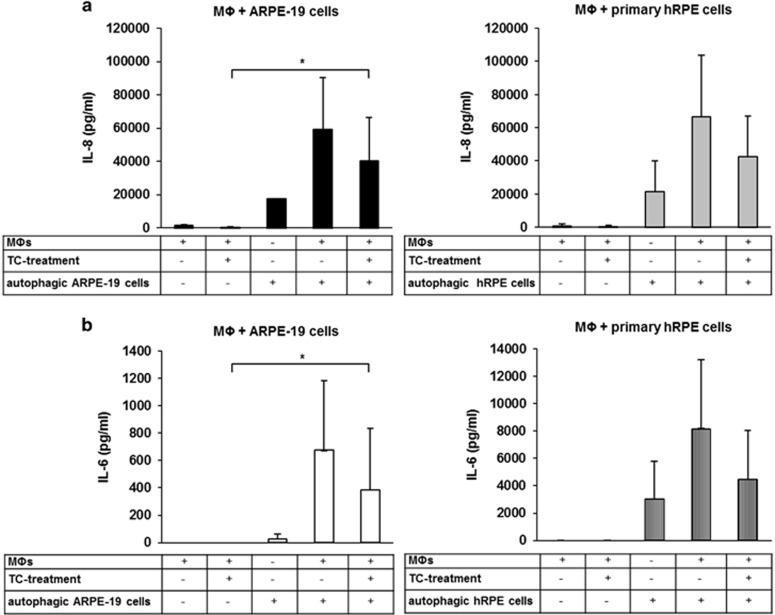
Release of IL-6 and IL-8 during phagocytosis of autophagy-associated dying RPE cells. H_2_O_2_-treated ARPE-19 (left panels) and hRPE cells (right panels) were co-incubated with untreated and TC-treated (48 h, 1 *μ*M) MФs for 8 h, then the supernatants were collected, and the concentration of IL-8 (**a**) and IL-6 (**b**) was determined by ELISA. Bars represent the mean±S.D. of three independent experiments, *P<0.05

The release of IL-6 and IL-8 during the clearance of autophagy-associated dying RPE cells by macrophages was studied next (Figure 7). Negligible amount of IL-6 and IL-8 secretion could be measured from TC-treated (48 h, 1 *μ*M) and untreated macrophages (in the absence of dying cells). H_2_O_2_-treated RPE cells themselves released significantly higher amount of IL-8 compared with the amount of secreted IL-6. Co-incubation of macrophages with the dying ARPE-19 or hRPE cells for 8 h lead to induction of a pro-inflammatory response as suggested by the increased levels of IL-6 and IL-8 measured in the culture supernatants. Furthermore, an increased amount of IL-8 was released when H_2_O_2_-induced dying cells were added to the macrophages compared with the released amount of IL-6. Owing to the anti-inflammatory effect of the glucocorticoid TC, the production of both IL-6 and IL-8 was decreased during engulfment of autophagy-associated dying RPE cells. Our data suggest that the clearance of autophagy-associated dying RPE cells by macrophages leads to a pro-inflammatory response *in vitro*. It is to note that the minority of the dead cells interacting with the macrophages were primary or secondary necrotic cells. Taken this fact into account, we cannot exclude their possible role in the induction of inflammatory cytokine release by macrophages completely.

## Discussion

Retinal cells can undergo a wide range of cell death modalities including apoptosis, anoikis, autophagy and necrosis throughout their lifetime. Several studies have demonstrated that autophagy decline or dysregulation is associated with AMD pathogenesis.^4, 37, 38, 39, 40, 53^ The molecular mechanisms controlling effective dead cells' clearance are poorly understood. Inefficient removal of dying cells by professional and non-professional phagocytes can result in the accumulation of cellular debris in the space between the Bruch's membrane and the RPE layer and development of AMD. Autophagy-related proteins have been detected in the drusen of eyes from patients suffering with the disease.^38^ In the current study, clearance of RPE cells undergoing autophagy-associated cell death by human macrophages, DCs and living RPEs was examined.

It is widely accepted that oxidative stress and production of reactive oxygen species in RPE cells have a major role in the pathogenesis of AMD. Increased levels of reactive oxygen species can lead to cellular or molecular damage and accumulation of detrimental products, for example, intracellular lipofuscin and extracellular drusen, which are a hallmark of age-related conditions. The imbalance between production of reactive oxygen species and antioxidant defense responses, such as catalase and superoxide dismutase activity, can result in an increased oxidative stress.^22, 54, 55^ It was described that reactive oxygen species can act as signaling molecules in nutrient starvation-induced autophagy, which has an important role in cellular survival response to stress conditions.^56, 57^ In healthy cells, autophagy is present at a basal level. However, hypoxia, oxidative stress and inflammation can enhance the accumulation of autophagic markers.^58, 59^

We observed an induction of autophagy in RPE cells after a 2h, 1 mM H_2_O_2_ treatment using TEM, immunoblotting for LC3/p62 expression and GFP-LC3 transfection assays. We could confirm and apply a recent finding that the fusion of AVs with lysosomes and degradation of autophagic proteins can be blocked by CQ treatment in ARPE-19 cells, which leads to increased levels of LC3-II.^48^ Combining these approaches, an *in vitro* detection model for autophagy in RPE cells could be established.

The continuous removal of dying cells from the tissues is essential for maintaining tissue homeostasis and physiological balance of the innate immunity.^25^ Autophagy contributes to programmed cell death and has a significant role in the exposure of energy-dependent ‘eat-me' signals, especially presentation of phosphatidylserine (PS) on the surface of dying cells.^60^ The uptake of autophagy-associated dying cells can be mediated by two different pathways: PS being exposed to the surface of dying cells for efficient recognition and removal by non-professional phagocytes,^61, 62^ and PS-independent engufment performed by macrophages acting as professional phagocytes.^26^ We observed that the percentage of PS-positive or dying RPE cells was increased in a time- and concentration-dependent manner upon H_2_O_2_ treatment: autophagy-associated cell death was induced as a result of serum deprivation and oxidative stress caused by H_2_O_2_ treatment in these cells. Nevertheless, the H_2_O_2_ treatment on RPE cells caused a mixture of different cell death modalities to be present at the same time.

RPE cells form the blood–retina barrier, the Bruch's membrane found underneath them being a regulator of the transport of biomolecules, oxygen, nutrients and metabolic waste products between the RPE and choriocapillaris.^63^ The apical membrane of the RPEs ensheathes the photoreceptors in the retina and engulfs the shed tips of the photoreceptor outer segments, thus recycling them on a daily basis.^61, 62^ RPEs are therefore one of the most effective or potent phagocytes in the human body. Phagocytosis by RPEs is responsible for the normal visual cycle, retinal homeostasis as well as support of normal photoreceptor function.^64, 65^

The pathogenesis of dry AMD is characterized by accumulation of dead cells, intracellular lysosomal lipofuscin and extracellular drusen deposits.^34^ In this case, the blood–retina barrier is intact, therefore, only non-professional phagocytes (living RPE cells) can engulf the dying neighboring cells. Our ARPE-19 cells engulfed efficiently and increasingly over time the autophagy-associated dying RPE *in vitro*.

In wet type of AMD, abnormal blood vessels penetrate through the blood–retinal barrier leading to hemorrhages and retinal edema. Many wet AMD studies have confirmed accumulation of macrophages in the drusen, in the areas of breakdown of Bruch's membrane and CNVs.^66^ Macrophages have a dual role in AMD: pro-inflammatory M1 macrophages can act as inflammatory stimulators, which might induce tissue damage, in contrast to the relatively anti-inflammatory M2 macrophages, which function as housekeepers and have a significant role in the clearance of drusen deposits.^67^ Moreover, the presence of DCs in drusen-associated changes in the retina has been reported recently in cases of RPE cells' injury.^68^

Mertk expression on the surface of macrophages has an important role in the clearance of dying cells. DCs also express Mertk, which seems not to be involved in this process. In addition, Axl and Tyro3 receptors are also necessary for the phagocytic activity and have a crucial role in DCs, but to a lesser extent in macrophages. In case of non-professional RPEs serving as phagocytes, Mertk is the key receptor for triggering ingestion.^69^ Non-professional and professional phagocytes have a major role in the pathogenesis of wet AMD.^33, 70^ In a recent study, we showed that MerTk has a key role in the regulation of TC-enhanced phagocytosis of RPE cells by non-professional and professional phagocytes.^41^ Here, we demonstrate that autophagy-associated dying RPE cells can be efficiently and increasingly engulfed by macrophages and DCs over time *in vitro.*

Corticosteroids such as TC have anti-inflammatory, anti-fibrotic and anti-angiogenic effects, as well as role in the stabilization of the blood–retinal barrier.^8^ TC treatment can transiently reduce the leakage from CNVs.^71^ In addition, significantly increased visual acuities after injections of TC have been ascribed previously.^72^ We have previously reported that TC treatment results in enhanced removal of anoikic dying RPEs *in vitro.*^33^ In line with this, we observed that TC treatment in macrophages can enhance the phagocytic uptake of autophagy-associated dying RPE cells.

The effect of 3-MA, a widely used inhibitor of autophagy and blocker of the autophagosome formation through inhibition of class III phosphatidylinositol 3-kinases, on the cell death and the clearance of H_2_O_2_-treated RPE cells was also studied here. 3-MA treatment could partially block the autophagic process, the subsequent cell death and cause decreased rate of phagocytosis of these RPE cells by macrophages. This finding is in line with our previously published results in which both death and phagocytosis could be inhibited by 3-MA in dying MCF-7 cells. These data suggest that autophagy contributes to the specific changes of the cell surface, which are associated with recognition and removal of these dying cells by phagocytes.^49^

The monitoring and quantifying methods of autophagy are limited because of the inconsistency in autophagic markers. LC3 protein is the key marker of the autophagic process in mammalian cells; its lipidated form is attached to the autophagosomal membrane. The most commonly used approaches to study the autophagic activity are the detection of the level of LC3 protein by western blot analysis or visualization of LC3-positive puncta by fluorescent microscopy as well as identification of autophagosomes by TEM.^47^ In addition, GFP-LC3, a fusion protein, has been widely used as an established autophagosomal marker for monitoring autophagic activity both biochemically and microscopically. Flow cytometry has recently been used to quantify the fluorescence intensity of GFP-LC3, which indicates the level of autophagy in the GFP-LC3-transfected cells. FACS analysis is a sensitive, simple, high-throughput technique that can be used to sort GFP-LC3-positive and -negative sub-populations of transfected cells based on their size, granularity or fluorescence signal.^73, 74^ In the present study, serum-deprived and H_2_O_2_ co-treated, GFP-LC3-labeled ARPE-19 cells expressed higher GFP fluorescence intensity compared with the untreated control cells. As H_2_O_2_ treatment of RPE cells results in a heterogenous cell population, we intended to exactly assess the uptake of pure autophagy-associated dying ARPE-19 cells by macrophages. To our present knowledge, this is the first study showing quantification and visualization of the clearance of H_2_O_2_-treated, GFP-LC3-positive sorted ARPE-19 cells by macrophages.

Inflammation has an essential role in many biological processes, such as protective responses to harmful stimuli, elimination of damaged tissues or preservation of normal tissue homeostasis. The eye functions as a immune-privileged site in the human body capable of inducing immune suppression. Defects in this mechanism can lead to the development of several ocular inflammatory processes, some of which may contribute to AMD pathogenesis.^75^

The release of cytokines from innate immune cells are crucial regulators of a pro- or anti-inflammatory response (IL-6, IL-8 and TNF-*α*). Failure to balance between different types of cytokines produced may also be associated with development of AMD. Recently, it has been shown that high levels of IL-6 in the blood could induce activation of pro-angiogenic growth factors, such as vascular endothelial growth factor, which is implicated in the progression of CNV and AMD as well.^37^ In the future, IL-6 may be a possible novel target for AMD therapy. A correlation between IL-8 polymorphism and AMD has also been shown, as well as contribution of IL-8 to angiogenesis, CNV and macular edema in AMD.^76, 77^ In this study, we showed a strong downregulative effect for interleukins' release by TC in autophagy-associated dying RPE cells.

Impaired heterophagy and autophagy in non-professional RPE cells are linked to the pathogenesis of AMD. To our knowledge, this is the first indication that RPE cells undergoing autophagy-associated cell death engage in clearance mechanisms guided by professional and non-professional phagocytes and accompanied by induction of inflammation in an *in vitro* model for AMD. We believe that not only intracellular protein clearance in RPE cells, but also clearance of autophagy-associated cell death debris by non-professional and professional phagocytes are essential in the pathology of AMD, and thus might serve as novel therapeutic target.

## Materials and Methods

### Ethics statement

Primary hRPE cells were isolated from human cadaver eyes under the auspices of a National Ethical Committee approval and following the Guidelines of the Declaration of Helsinki.

Buffy coats were provided anonymously by the Hungarian National Blood Service where blood was taken from healthy volunteers and written informed consent from all participants was obtained. For these studies, approval was obtained from the ethics committee of the Medical and Health Science Center, University of Debrecen (DEOEC RKEB/IKEB Prot. No. 2745 -2008 and 3093 - 2010). The ethics committee approved this consent procedure.

### Cell culture and treatments

Dulbecco's modified Eagle's medium (DMEM) (Sigma-Aldrich, St. Louis, MO, USA), DMEM Nutrient mixture F12 (Sigma-Aldrich), H_2_O_2_ (Sigma-Aldrich), CQ (Sigma-Aldrich), 3-MA (Sigma-Aldrich), TC (Sigma-Aldrich), phosphate-buffered saline (PBS) (HyClone, Logan, UT, USA), carboxyfluoresceindiacetate-succinimidyl ester (CFDA) (Molecular Probes, Eugene, OR, USA), 5-(and-6)-(((4-chloromethyl)benzoyl) amino)tetramethylrhodamine (CMTMR) (Molecular Probes), plastic tissue culture flasks (TPP, Trasadingen, Switzerland) were used in this work.

ARPE-19, a human RPE cell line, was kindly provided by Prof. Stephen Moss, (UCL, London, UK) and was cultured at 37 °C, 5% CO_2_ in DMEM supplemented with 10% fetal calf serum (Gibco, Paisley, UK), 200 mM L-glutamine (Sigma-Aldrich) and 1% antibiotic/antimycotic solution (HyClone). The experiments were performed on passage 10–15 ARPE-19 cells.

The primary hRPE cells were obtained from five different adult cadaver human eyes (age range: 64–92) without any known ocular diseases. hRPE cells were isolated from cadavers after removal of the anterior segment (corneo-scleral ring) and the lens, then paper sponges and forceps were used to remove the vitreous and neuroretina, respectively. Consequently, half-spherically bent-end Pasteur glass pipettes were used to gently scape the RPE layer without damaging the Bruch's membrane and the collected cell suspension placed in PBS for centrifugation (10 min, 1000 r.p.m.), then cultured in DMEM Nutrient mixture F12 medium supplemented with 10% fetal calf serum, 200 mM L-glutamine and 1% antibiotic/antimycotic solution. The hRPE cells were used at passages 2–5 for all experiments.

Cells were detached from the cell culture flasks and plates using trypsin/ethylenediaminetetraacetic acid (Sigma-Aldrich). For the induction of autophagy, ARPE-19 and primary hRPE cells were plated over a 24h period and cultured until they formed confluent (80–90%) monolayers, then treated by 0.4–1 mM H_2_O_2_ for 2–4 h before harvesting. ARPE-19 cells were pre-treated with 10 mM 3-MA for 24 h for inhibition of autophagy.^27^

### Assays of cell death

H_2_O_2_-induced cell death was assessed by the Annexin V-fluorescein isothiocyanate Apoptosis Detection Kit (MBL, Woburn, MA, USA) according to the manufacturer's recommendations. AnxV-FITC/PI staining was used to determine the rate of PS^+^ cells and plasma membrane permeability. Percent of cells positive for AnxV or PI was determined by relative fluorescence intensity using a BD FACSCalibur flow cytometer (BD Biosciences, San Jose, CA, USA).^49^

### Quantification of LC3-positive cells by FACS analysis and fluorescence microscopy

Autophagy was assessed by detection of AVs in GFP-LC3-transfected ARPE-19 cells. ARPE-19 cells were grown on adherent glass coverslips before they were transiently transfected with a GFP-LC3 expression plasmid kindly provided by Prof. Noboru Mizushima (Tokyo Medical and Dental University, Tokyo, Japan), using polyethylenimine (PEI) reagent (Sigma-Aldrich) (PEI:DNS ratio=4 : 1, 1 *μ*g DNA/well). Transfected ARPE-19 cells were incubated with 25 *μ*M CQ for 1 h and then treated with 1mM H_2_O_2_ for 2 h in serum-free medium. Coverslips were fixed in 4% paraformaldehyde (Sigma-Aldrich) for 10 min and stained with 2-(4-amidinophenyl)-6-indolecarbamidine dihydrochloride (DAPI) (0.3 *μ*g/ml) (Sigma-Aldrich) to visualize cell nuclei. Images were taken by an Axiovert-200 Zeiss microscope (Carl Zeiss MicroImaging GmbH, Göttingen, Germany). The number of GFP-LC3-positive cells were counted manually or quantified by flow cytometry using a BD FACS Aria III (BD Biosciences).

In order to detect AVs and cell death in ARPE-19 cells simultaneously, transient transfection of mCherry-LC3 plasmid, which was kindly provided by Dr. Gian Maria Fimia (University of Salento, Lecce, Italy), was completed. These transfected cells were treated with 1 mM H_2_O_2_ for 2 h. PS externalization, which reflects upon apoptotic cell death, was determined by Annexin V-FITC labeling. Cells containing LC3-positive AVs and/or cells exposing PS on their surface were quantified using a BD FACSCalibur flow cytometer.

GFP-LC3-positive ARPE-19 cells were sorted out on the basis of their GFP fluorescence. Cells were harvested by trypsinization, washed, centrifugated and resuspended in PBS to a final density of 2 × 10^6^ cells/ml, and filtered through a nylon filter (Merck-Millipore, Darmstadt, Germany) to remove cell aggregates. Flow cytometry and cell sorting for GFP fluorescence were performed using a BD FACS Aria III. Data acquisition and analysis were performed using BD FACS Diva 6.2 software. GFP signals were detected with a 530/30-nm bandpass filter. The GFP-LC3-positive, AV-containing cells and parallelly the GFP-LC3-negative cells were selected by gates and the fluorescence intensity of events within the gated regions was quantified. Data were collected from 10 000–20 000 events for each sample. Control-sort was performed to prove a greater than 98% sorting efficiency. The green fluorescent cell population of interest was gated based on relative fluorescence intensity.^78^

### Electron microscopy

Samples were fixed in 0.1 M sodium cacodylate-buffered, pH 7.4 and 2.5% glutaraldehyde solution for 2 h and then rinsed (three times, 10 min) in 0.1 M sodium cacodylate buffer, pH 7.4 and 7.5% saccharose and postfixed in 1% OsO_4_ solution for 1 h. After dehydration in an ethanol gradient (70% ethanol (20 min), 96% ethanol (20 min), 100% ethanol (two times, 20 min)), samples were embedded in Durcupan ACM. Ultrathin sections were stained with uranyl acetate and lead citrate. Sections were examined in a Philips CM 10 microscope (Philips Electronic Instruments, Mahwah, NJ, USA) at 80 kV.^33^

### Antibodies and immunoblotting

An anti-LC3 rat polyclonal antibody (Novus Biologicals, Littleton, CO, USA), which recognizes both LC3-I and LC3-II and an anti-p62 mouse monoclonal antibody (Santa Cruz Biotechnology, Dallas, TX, USA) were used to detect autophagy. Cells were collected and washed with PBS, suspended in lysis buffer (50 mM Tris–HCl; 0.1% Triton X-100 (Sigma-Aldrich); 1 mM ethylenediaminetetraacetic acid (Sigma-Aldrich); 15 mM 2-mercaptoethanol (Sigma-Aldrich) and protease inhibitor (Sigma-Aldrich). Insoluble cellular material was removed by centrifugation and the lysates were mixed with 5 × Laemmli loading buffer, boiled for 10 min. Equal amounts of protein (20 *μ*g) were separated on 15% SDS-polyacrylamide gel, and transferred onto a PVDF Immobilon-P Transfer Membrane (Merck-Millipore; pore size 0.45 *μ*m). The membranes were blocked in Tris-buffered saline containing 0.05% Tween-20 (Sigma-Aldrich) (TBS-T) and 5% skimmed milk (AppliChem, Darmstadt, Germany) for 1 h. Then, membranes were probed overnight at 4 °C with anti-LC3 (1 : 2000), anti-p62 (1 : 2000), anti-tubulin (1 : 5000) (Sigma-Aldrich), anti-GAPDH (1 : 5000) (Covalab, Villeurbanne, France) antibody in TBS-T containing 1% nonfat skimmed milk, followed by incubation with horseradish-peroxidase-conjugated species corresponding secondary antibodies (Sigma-Aldrich) for 1 h at room temperature. Immunoreactive proteins were visualized using Immobilon Western chemiluminescence substrate (Millipore-Merck). Densitometry was carried out using the ImageJ software.

### Phagocytosis assay

Human monocytes were isolated from ‘buffy coats' of healthy blood donors by Ficoll–Paque Plus (Amersham Biosciences, Piscataway, NJ, USA) gradient and magnetic separation using CD14 human MicroBeads (MiltenyiBiotec, BergischGladbach, Germany). Human macrophages were obtained through a 5-day differentiation using 5 ng/ml macrophage colony-stimulating factor (MCSF) (Peprotech EC, London, Great Britain) at 37 °C in Iscove's Modified Dulbecco's Medium (IMDM) (Gibco) containing 10% human AB serum (Sigma-Aldrich) and 10000 U/ml penicillin and 10 mg/ml streptomycin (Sigma-Aldrich).^41^ To differentiate iDCs, monocytes were plated into 6-well culture dishes at a density of 2 × 10^6^ cells/ml and cultured for 5 days in serum-free AIM V medium (Gibco) containing 80 ng/ml GMCSF (Peprotech EC) and 100 ng/ml IL-4 (Peprotech EC). On day 2, the same amounts of GMCSF and IL-4 were added to the cell cultures without changing their media for another 3 days.^79, 80^ Resting DCs were activated on day 5 by inflammatory cytokine mixture containing 10 ng/ml TNF-*α* (Peprotech EC), 5 ng/ml IL-1β (Peprotech EC), 20 ng/ml IL-6 (Peprotech EC), 75 ng/ml GMCSF (Peprotech EC) and 1 mg/ml prostaglandin E2 (PGE_2_) (Sigma-Aldrich) and harvested on day 6.^52, 81^ Monocyte-to-DC differentiation was controlled by the phenotypic analysis using anti-CD209 (R&D Systems, Minneapolis, MN, USA), anti-CD83 (R&D Systems) and anti-CD86 (R&D Systems) antibodies. Living RPE cells acting as phagocytes were plated in serum-free medium 24 h before phagocytosis. Phagocytes were pre-treated with 1 *μ*M TC 48 h prior to the assay.^33, 41^ Dying RPE cells were fed to engulfing cells following the induction of autophagy-associated cell death by 1 mM H_2_O_2_ treatment for 2 h. Engulfing cells were stained for 16 h with 7.5 *μ*M CMTMR, while the dying cells were labeled for 2 h with 12.5 *μ*M CFDA-SE followed by washing twice with PBS before phagocytosis. Phagocytes and dying RPE cells were mixed at a ratio of 1 : 3 in the absence of human 10% AB serum and incubated for 4, 8, 12 or 24 h at 37 °C, 5% CO_2_ atmosphere. The whole-cell mixture was collected by trypsin digestion to remove bound but not engulfed dying cells, centrifuging, washing twice in PBS and fixing in 1% PBS-buffered paraformaldehyde (pH 7.4). The phagocytosis rate was determined by FACS analysis as percent phagocytic cells (CMTMR positive) that have engulfed dying cells (positive for both CMTMR and CFDA-SE).^49^ In addition, the GFP-LC3-positive as well as the negative sorted ARPE-19 cells were resuspended in IMDM and added to the macrophages. The rate of phagocytosis was analyzed after 8 h co-incubation.

### Time-lapse imaging microscopy

For *in vitro* phagocytosis assay, the dying RPE cells were stained with CFDA-SE and co-incubated with the CMTMR-stained macrophages in a ratio of 2 : 1. For the time-lapse microscopy of the co-cultures, an incubation chamber system (Solent Scientific, Segensworth, UK) attached to a motorized Olympus IX-81 inverted microscope (Olympus Europa Holding, Hamburg, Germany) equipped with a cooled high-speed Hamamatsu ORCA-R2 camera (HamamatsuPhotonics, Hamamatsu City, Japan) was used. The incubation chamber system consisted of a temperature logging controller (consistent 37 °C), a sterile air flow and humidity circulator and an inner CO_2_ enrichment multi-well plate holder. Cells were cultured on 24-well cell culture plates. Images were taken automatically for 24 h through a PlasDIC filter in every 5 min per channel and per well with the help of a motorized cubic filter. The time-lapse video was created from the digital images with the use of the XCE-RT xCellence Real Time software with 24 fps (Olympus).^82^

### Quantification of IL-6 and IL-8 release by ELISA

Differentiated macrophages were co-incubated with H_2_O_2_-treated (2 h, 1 mM) ARPE-19 and hRPE cells for 8 h, and the supernatants were collected for cytokine measurements. Macrophages were either treated with 1 *μ*M TC for 48 h or left untreated prior to starting the phagocytosis assays. The concentration of IL-6 (pg/ml) and IL-8 (pg/ml) was measured from the collected cell culture media using Human IL-6 ELISA OptEIA kits (BD Biosciences) and Human IL-8 ELISA OptEIA kits (BD Biosciences) according to the manufacturer's instructions.

### Statistical analysis

Results are expressed as the mean±S.D. or mean±S.E.M. for the number of assays indicated. For multiple comparisons of groups, statistical significance was calculated and evaluated by one-way ANOVA followed by Tukey *post hoc* test. For comparison of two groups, Student's *t*-test was used. *P*-values <0.05 were considered statistically significant.

## Abbreviations

RPE: retinal pigment epithelium
AMD: age-related macular degeneration
DC: dendritic cell
H_2_O_2_: hydrogen peroxide
LC3: light-chain 3
CQ: chloroquine
3-MA: 3-methyladenine
TC: triamcinolone
CNV: choroidal neovascularization
AV: autophagic vacuole
TEM: transmission electron microscopy
GFP: green fluorescent protein
FACS: fluorescence-activated cell sorter
AnxV: annexin V
PI: propidium iodide
GMCSF: granulocyte-macrophage colony-stimulating factor
PS: phosphatidylserine
DMEM: Dulbecco's modified Eagle's medium
CFDA-SE: carboxyfluorescein-diacetate-succinimidyl ester
CMTMR: 5-(and-6)-(((4-chloromethyl)benzoyl) amino)tetramethylrhodamine
PEI: polyethylenimine
DAPI: 2-(4-amidinophenyl)-6-indolecarbamidine dihydrochloride
MCSF: macrophage colony-stimulating factor
IMDM: Iscove's Modified Dulbecco's Medium
