# Supplementary material for: Clearance of autophagy-associated dying retinal pigment epithelial cells – a possible source for inflammation in age-related macular degeneration
Source: Cell Death Dis. 2016 Sep 8;7(9):e2367–. doi: 10.1038/cddis.2016.133 (PMC5059849; doi:10.1038/cddis.2016.133)
Supplement: Supplementary Information [file cddis2016133x1.doc]

**Supplementary video 1. Time-lapse analysis of phagocytosis of autophagy-associated dying ARPE-19 cells by macrophages (MΦs).**

MΦs (CMTMR stained) were co-incubated with H_2_O_2_-treated (2h, 1mM) ARPE-19 cells (CFDA-stained) in a ratio of 1:2 for 24h. Images were taken every five minutes by time-lapse microscopy, which were converted to video with the use of the Xcellence software. The engulfment of autophagy-associated dying ARPE-19 cells by MΦs was observed over time. The videos are representative of three different experiments.

**Supplementary video 2. Time-lapse analysis of phagocytosis of autophagy-associated dying ARPE-19 cells by TC-treated MΦs.**

TC pre-treated MΦs (48h, 1µM) (CMTMR stained) were co-incubated with H_2_O_2_-treated (2h, 1mM) ARPE-19 cells (CFDA stained) in a ratio of 1:2 for 24h. Images were taken every five minutes by time-lapse microscopy, which were converted to video with the use of the Xcellence software. The engulfment of autophagy-associated dying ARPE-19 cells by TC-treated MΦs was observed over time. The videos are representative of 3 different experiments.

**Supplementary video 3. Time-lapse analysis of clearance of autophagy-associated dying primary hRPE cells by MΦs.**

MΦs (CMTMR stained) were co-incubated with H_2_O_2_-treated (2h, 1mM) hRPE cells (CFDA stained) in a ratio of 1:2 for 24h. Images were taken every five minutes by time-lapse microscopy, which were converted to video with the use of the Xcellence software. The clearance of autophagy-associated dying hRPE cells by MΦs was observed over time. The videos are representative of 3 different experiments.

**Supplementary video 4. Time-lapse analysis of clearance of autophagy-associated dying primary hRPE cells by TC-treated MΦs.**

TC pre-treated MΦs (48h, 1µM) (CMTMR stained) were co-incubated with H_2_O_2_-treated (2h, 1mM) hRPE cells (CFDA stained) in a ratio of 1:2 for 24h. Images were taken every five minutes by time-lapse microscopy, which were converted to video with the use of the Xcellence software. The clearance of autophagy-associated dying hRPE cells by TC-treated MΦs was observed over time. The videos are representative of 3 different experiments.

**Supplementary video 5. Time-lapse analysis of the engulfment of GFP-LC3 transfected, H_2_O_2_-treated ARPE-19 cells by MΦs.**

MΦs (CMTMR stained) were co-incubated with GFP-LC3 transfected, H_2_O_2_-treated (2h, 1mM) ARPE-19 cells in a ratio of 1:2 for 24h. Images were taken every five minutes by time-lapse microscopy, which were converted to video with the use of the Xcellence software. The removal of GFP-LC3 transfected, H_2_O_2_-treated ARPE-19 cells by MΦs was observed over time. The videos are representative of 3 different experiments.

**Supplementary video 6. Time-lapse analysis of engulfment of GFP-LC3 transfected, H_2_O_2_-treated ARPE-19 cells by TC-treated MΦs.**

TC pre-treated MΦs (48h, 1µM) (CMTMR stained) were co-incubated with GFP-LC3 transfected, H_2_O_2_-treated (2h, 1mM) ARPE-19 cells in a ratio of 1:2 for 24h. Images were taken every five minutes by time-lapse microscopy, which were converted to video with the use of the Xcellence software. TC treatment significantly enhanced the engulfment of GFP-LC3 transfected, H_2_O_2_-treated ARPE-19 cells by MΦs**.** The videos are representative of 3 different experiments.

**Supplementary video 7. Time-lapse analysis of the clearance of sorted GFP-LC3 positive, H_2_O_2_-treated ARPE-19 cells by MΦs.**

MΦs (CMTMR stained) were co-incubated with sorted GFP-LC3 positive, H_2_O_2_-treated (2h, 1mM) ARPE-19 cells in a ratio of 1:2 for 24h. Images were taken every five minutes by time-lapse microscopy, which were converted to video with the use of the Xcellence software. MΦs were able to efficiently engulf sorted GFP-LC3 positive ARPE-19 cells. The videos are representative of 3 different experiments.
