# Supplementary material for: Clearance of autophagy-associated dying retinal pigment epithelial cells – a possible source for inflammation in age-related macular degeneration
Source: Cell Death Dis. 2016 Sep 8;7(9):e2367–. doi: 10.1038/cddis.2016.133 (PMC5059849; doi:10.1038/cddis.2016.133)
Supplement: Supplementary Figure 1 [file cddis2016133x9.pdf]

SUPPLEMENTARY FIGURE

Clearance of autophagy-associated dying retinal pigment epithelial cells - a possible source for inflammation in age-related macular degeneration

*Mária Szatmári-Tóth, Endre Kristóf, Zoltán Veréb, Saeed Akhtar, Andrea Facskó, László Fésüs, Anu Kauppinen, Kai Kaarniranta, Goran Petrovski**

*Corresponding author

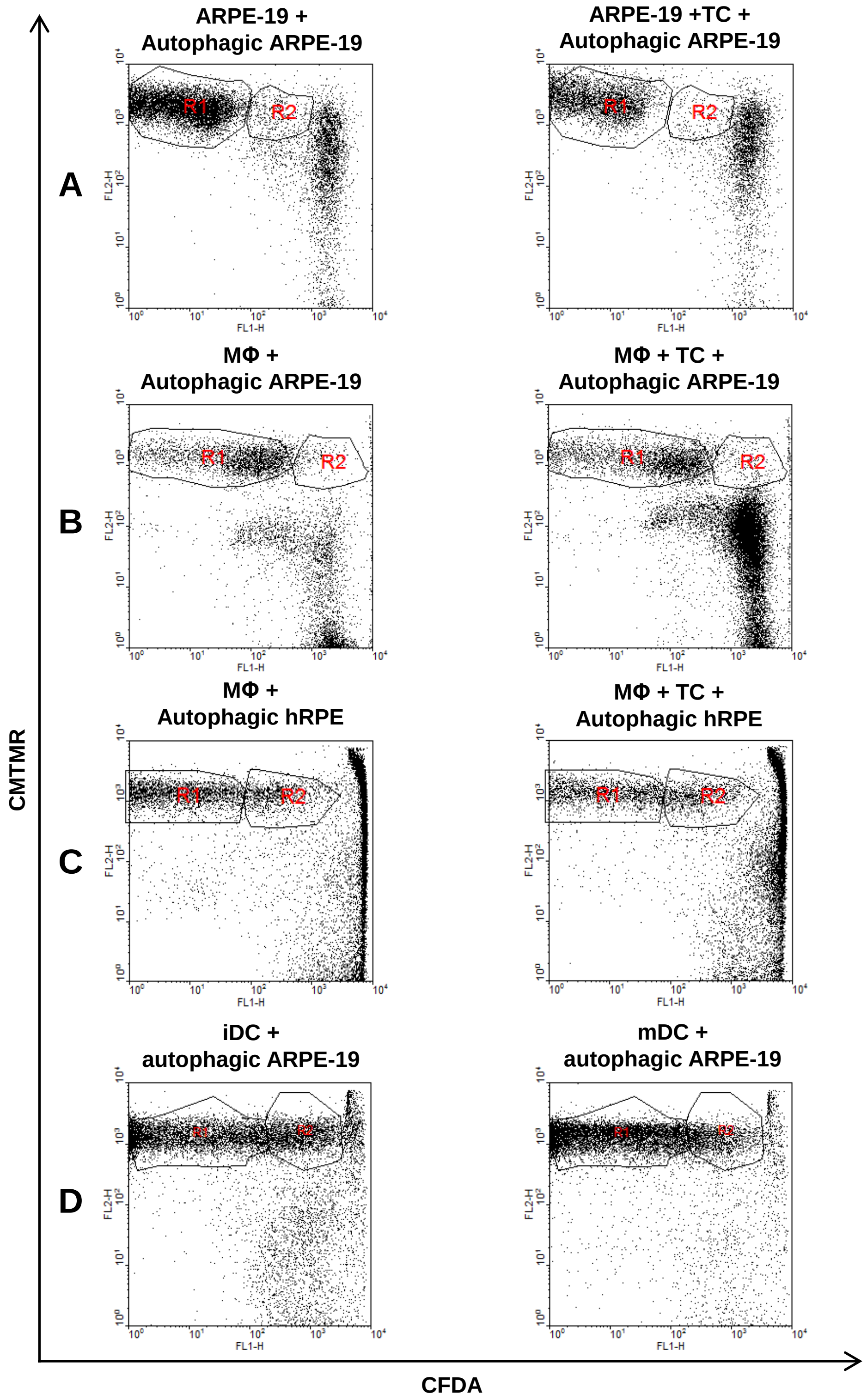

Supplementary Figure 1. Representative flow cytometry dot plots demonstrating phagocytosis of* autophagy-associated dying *RPE cells by non-professional and professional phagocytes in vitro. The clearance of autophagy-associated dying ARPE-19 cells (CFDA-labeled) by living ARPE-19 cells (A) and macrophages (MΦ) (B) (CMTMR-labeled) after 8h co-incubation is shown as determined by FACS analysis. Phagocytes were pre-treated with 1µM triamcinolone (TC) for 48h. (C) The phagocytosis rate of autophagy-associated dying hRPE cells (CFDA-labeled) by untreated and TC-pretreated (48h, 1µM) macrophages (CMTMR-labeled) were measured after 8h co-incubation by FACS analysis. (D) Phagocytic capacity of immature dendritic cells (iDCs) and mature dendritic cells (mDCs) (CMTMR-labeled) for engulfment of autophagy-associated dying ARPE-19 cells (CFDA-labeled) after 8h co-incubation is shown. The horizontal axis represents the intensity of staining for CFDA (logarithmic scale) and the vertical axis shows the intensity of staining for CMTMR (logarithmic scale). The cells within the „R2" designated area represent the engulfed RPE (CFDA-labeled) cells by non-professional and professional phagocytes (CMTMR-labeled). „R1" area contains the phagocytes which did not engulf dying cells. Data are representative of 4 independent experiments.
